# ESR1 as a recurrence-related gene in intrahepatic cholangiocarcinoma: a weighted gene coexpression network analysis

**DOI:** 10.1186/s12935-021-01929-5

**Published:** 2021-04-17

**Authors:** Fengwei Li, Qinjunjie Chen, Yang Yang, Meihui Li, Lei Zhang, Zhenlin Yan, Junjie Zhang, Kui Wang

**Affiliations:** 1 Department of Hepatic Surgery (II) of the Eastern Hepatobiliary Surgery Hospital, Navy Medical University, (Second Military Medical University), #225 Changhai Road, Shanghai, 200438 China; 2 Department of Hepatic Surgery (IV) of the Eastern Hepatobiliary Surgery Hospital, Navy Medical University, Shanghai, China; 3 Department of Hepatic Surgery (VI) of the Eastern Hepatobiliary Surgery Hospital, Navy Medical University, Shanghai, China; 4 Department of Obstetrics and Gynecology, Changhai Hospital, Naval Military Medical University, #168, Changhai Road, Yangpu District, Shanghai, 200433 China

**Keywords:** Intrahepatic cholangiocarcinoma, ESR1, Weighted gene coexpression network analysis, Recurrence

## Abstract

**Background:**

Intrahepatic cholangiocarcinoma (iCCA) is the second most common malignant hepatic tumor and has a high postoperative recurrence rate and a poor prognosis. The key roles of most tumor recurrence-associated molecules in iCCA remain unclear. This study aimed to explore hub genes related to the postsurgical recurrence of iCCA.

**Method:**

Differentially expressed genes (DEGs) between iCCA samples and normal liver samples were screened from The Cancer Genome Atlas (TCGA) database and used to construct a weighted gene coexpression network. Module-trait correlations were calculated to identify the key module related to recurrence in iCCA patients. Genes in the key module were subjected to functional enrichment analysis, and candidate hub genes were filtered through coexpression and protein–protein interaction (PPI) network analysis. Validation studies were conducted to detect the “real” hub gene. Furthermore, the biological functions and the underlying mechanism of the real hub gene in iCCA tumorigenesis and progression were determined via in vitro experiments.

**Results:**

A total of 1019 DEGs were filtered and used to construct four coexpression modules. The red module, which showed the highest correlations with the recurrence status, family history, and day to death of patients, was identified as the key module. Gene Ontology (GO) enrichment and Kyoto Encyclopedia of Genes and Genomes (KEGG) pathway analyses demonstrated that genes in the red module were enriched in genes and pathways related to tumorigenesis and tumor progression. We performed validation studies and identified estrogen receptor 1 (ESR1), which significantly impacted the prognosis of iCCA patients, as the real hub gene related to the recurrence of iCCA. The in vitro experiments demonstrated that ESR1 overexpression significantly suppressed cell proliferation, migration, and invasion, whereas ESR1 knockdown elicited opposite effects. Further investigation into the mechanism demonstrated that ESR1 acts as a tumor suppressor by inhibiting the JAK/STAT3 signaling pathway.

**Conclusions:**

ESR1 was identified as the real hub gene related to the recurrence of iCCA that plays a critical tumor suppressor role in iCCA progression. ESR1 significantly impacts the prognosis of iCCA patients and markedly suppresses cholangiocarcinoma cell proliferation, migration and invasion by inhibiting JAK/STAT3 signaling pathway.

**Supplementary Information:**

The online version contains supplementary material available at 10.1186/s12935-021-01929-5.

## Background

Intrahepatic cholangiocarcinoma (iCCA) is the second most common malignant hepatic tumor after hepatocellular carcinoma (HCC) [1–3]. iCCA is a devastating disease with a poor prognosis and a high postoperative recurrence rate of 40–80% [3, 4]. Fewer than one-third of iCCA patients who undergo curative-intent surgery survive beyond 5 years after surgery [4, 5]. The age-specific mortality rate of this disease has increased in almost all countries across all continents, albeit at different rates [3]. The rising incidence and high mortality of iCCA have aroused growing worldwide concerns, especially over the last two decades [6]. The significant increase in the mortality rate of this primary hepatobiliary cancer coincides with the rapidly growing interest of clinicians and investigators [3, 7]. It is therefore an urgent task to clarify the mechanisms underlying the rapid progression of iCCA and identify novel molecular biomarkers for the recurrence and prognosis of this disease. Risk factors contributing to the prognosis of iCCA include cirrhosis, chronic viral hepatitis, excess alcohol use, diabetes, and obesity. The similarities between the risk factors of iCCA and HCC suggest that common pathobiological pathways may exist in all primary liver parenchymal tumors [8]. Numerous genetic alterations occur during iCCA development and progression. These alterations include mutational activation involving IDH1/2, KRAS, BRAS, and EGFR; mutational inactivation involving ARID1A, BAP1, and PBRM1; and changes in tyrosine kinase (TK) fusion proteins, including FGFR2 and ROS [2, 9, 10]. Tumor development and progression are complex and involve progressive and multifactorial processes [11]. However, the molecular pathogenesis of iCCA remains unclear.

With the discovery and development of high-throughput data analysis, screening for key information has become the basis for subsequent research. Weighted gene coexpression network analysis (WGCNA) is an effective bioinformatics strategy commonly used to explore the complex relationships between genes and related clinical traits. This method has been increasingly used in bioinformatics to analyze gene expression microarray profiles [12–14]. Genes that are highly coexpressed with other genes are connected in networks and then grouped into modules. Each module exhibits highly connected network regions, and genes in the same module may share similar biological regulatory functions. The relationship between modules and clinical traits can also be analyzed. Hub genes in hub modules are potential biomarkers for prognosis or therapy and can thus be selected for further validation.

In this study, we used WGCNA for the first time to analyze the clinical traits and mRNA expression profiles of iCCA patients obtained from The Cancer Genome Atlas (TCGA) database in an attempt to identify hub genes associated with iCCA recurrence after surgery. Our research determined that ESR1 plays a critical tumor suppressor role in iCCA. iCCA patients with low ESR1 expression had a poorer prognosis than those with high ESR1 expression. These findings could be beneficial for the assessment of the malignant potential of iCCA and provide therapeutic methods for this cancer.

## Materials and methods

### Data and tissue collections

The mRNA expression profiles and related clinical traits of iCCA patients were obtained from the TCGA database (https://cancergenome.nih.gov/). The TCGA–CHOL project consists of seven hilar/perihilar cholangiocarcinoma samples, two distal cholangiocarcinoma samples, 30 iCCA samples, and eight adjacent normal samples. The RNA-Seq data of 30 iCCA samples and eight corresponding adjacent normal samples were selected for analysis. Another 30 paired iCCA tumor tissues and normal liver tissues were collected from Eastern Hepatobiliary Surgery Hospital as the validation cohort. Informed consent was obtained from all the patients, and all procedures performed were in accordance with the Helsinki Declaration of 1975.

### Screening for differentially expressed genes (DEGs)

DEGs between iCCA samples and adjacent normal samples were screened using the “DESeq2” R package, in which the thresholds were set to false discovery rate (FDR) < 0.01 and |log2FoldChange|> 1.0.

### WGCNA construction

The quality of the RNA-Seq data was evaluated before the construction of the weighted coexpression network. Samples were deemed defective if they were distant from the majority in clustering by average linkage (defective sample = 0.75). The remaining samples were selected for follow-up analysis. The “WGCNA” package in R was used to construct a scale-free coexpression network for the DEGs that met the screening criteria. We considered that selection of the appropriate soft-thresholding power for a scale-free coexpression network is crucial and separately evaluating thresholding powers from 1 to 10 to identify the most appropriate value. Adjacencies among all DEGs were calculated using the selected power function. The adjacency matrix was subsequently transformed into a topological overlap matrix (TOM) to calculate dissimilarity (1-TOM). Modules were identified through a dynamic tree cut method, and the minimum size of each module was set to 30 genes. MEDissTres was set as 0.24 to merge similar modules.

### Identification of significant clinical modules

Gene significance (GS), module significance (MS) and the module eigengene (ME) were assessed. GS was defined as the log_10_ transformation of the *P*-value in the linear regression between gene expression and clinical traits (GS = lg*P*). MS was defined as the mean GS for all the genes in a module. The ME was defined as the first principal component in the principal component analysis for a given gene module. The module with the maximum absolute MS among all modules is generally regarded as related to a clinical trait. In addition, the correlation between the MEs and clinical traits was calculated. The module with the highest absolute MS was considered the key module related to a given clinical trait and was selected for further analysis.

### Functional enrichment analysis of the key modules

The Database for Annotation, Visualization, and Integrated Discovery (DAVID, https://david.ncifcrf.gov/summary.jsp) was used for GO enrichment analysis. The gene list for the key module was uploaded to DAVID, and Gene Ontology (GO) enrichment results and Kyoto Encyclopedia of Genes and Genomes (KEGG) pathways were obtained. A *P*-value < 0.05 was deemed statistically significant.

### Identification of candidate hub genes

The hub genes in the hub module were initially selected using the following criteria: absolute value of GS for an interesting clinical trait > 0.2 and module membership in the hub module > 0.8. The genes screened with these criteria comprised the *Color_Hub* gene list. All genes from the hub module (*P*-value of GS < 0.05, *P*-value of MM < 0.05) were simultaneously uploaded to the Search Tool for the Retrieval of Interacting Genes database (STRING). A protein–protein interaction (PPI) network was constructed based on a minimum required interaction confidence score > 0.4. The result of the PPI network analysis was downloaded and visualized again using Cytoscape software. The hub genes in the PPI network were screened using the *cytoHubba* [15] application in Cytoscape software, which was designed to identify key nodes in networks. The key genes were screened using *cytoHubba* to generate the *PPI_Hub* gene list. Genes common to the *Color_Hub* and *PPI_Hub* gene lists were regarded as candidate hub genes for further validation and analysis.

### Validation of real hub genes

Thirty pairs of iCCA and adjacent nontumor tissues were obtained from the Eastern Hepatobiliary Surgery Hospital (Shanghai, China). The mRNA expression levels of the screened candidate hub genes were validated by qRT-PCR. In addition, differences in the protein levels of the candidate genes between iCCA specimens and normal liver specimens were investigated by immunohistochemistry (IHC). Finally, the candidate hub genes that significantly influenced the disease-free survival (DFS) of patients were selected as the “real” hub genes.

### Western blotting

Protein was extracted from cells with RIPA lysis buffer supplemented with a protease inhibitor. Protein extracts (30 μg) were subjected to 4–20% gradient SDS/PAGE and blotted onto a nitrocellulose (NC) membrane. The membrane was then blocked with 5% nonfat milk in TBS (Sigma, T8793), incubated with the primary antibody overnight at 4 °C and incubated with the secondary antibody; then, the membrane was washed with TBST three times, and finally visualized using an enhanced chemiluminescence (ECL) detection kit (Thermo Fisher Scientific, USA). Quantification was performed with ImageJ (Version 2.0.0).

### Immunohistochemical staining

For IHC analysis, tissue microarray (TMA) sections were incubated with anti-ESR1 antibody (1:400 dilution). IHC staining was scored by two independent pathologists who were blinded to the clinical characteristics of the patients. The scoring system was based on the intensity and extent of staining. Staining intensity was classified as 0 (negative), 1 (weak), 2 (moderate), or 3 (strong); staining extent was dependent on the percentage of positive cells (among 200 assessed cells) and was classified as 0 (< 5%), 1 (5–25%), 2 (26–50%), 3 (51–75%), or 4 (> 75%). According to the staining intensity and staining extent scores, the IHC result was classified as 0–1, negative (−); 2–4, weakly positive ( +); 5–8, moderately positive (+ +), and 9–12, strongly positive (+ + +).

### Cell culture

Cells were cultured at 37 °C with a 5% CO_2_ atmosphere. HiBEpiC, RBE, HCCC-9810 and FRH0201 cells were cultured in DMEM (Gibco, USA) supplemented with 10% FBS (Gibco), and QBC939 cells were cultured in RPMI 1640 (Gibco) supplemented with 10% FBS (Gibco). At 80–90% confluence, the cells were trypsinized and replated at 5 × 10^5^ cells/75 cm^2^.

### Cell transfection

We generated an ESR1-overexpressing HCCC-9810 cell line using a lentiviral system and an ESR1-knockdown RBE cell line using siRNAs targeting ESR1. The corresponding negative control cell lines were also generated using an empty lentivirus or a nontargeting control siRNA. Our cell transfection experiments were performed using Lipofectamine 3000 (Invitrogen, USA) according to the manufacturer’s instructions. The efficiency of cell transfection was determined by Western blotting after 48 h.

### Cell proliferation assay

Cell proliferation ability was investigated using the Cell Counting Kit-8 (CCK-8; Beyotime Institute of Biotechnology, Shanghai, China) assay. Approximately 5000 cells were plated in 96-well plates for 12 h. Then, the supernatant was removed, and CCK-8 solution (Thermo Fisher Scientific) was added to each well and further incubated for 24, 48, and 72 h at 37 °C. Finally, the absorbance of each well at 450 nm was detected by a spectrophotometer (Thermo Fisher Scientific, Waltham, USA).

### Colony formation assay

Cells at a density of 200 cells/well were plated in six-well plates. After culturing for 14 days, the generated colonies were fixed with methanol for 15 min, stained with a hematoxylin solution and photographed using a microscope (Olympus, Japan). The assays were conducted in triplicate.

### Cell migration and invasion assay

Cell migration and invasion assays were performed using Transwell chambers (Corning, USA). Approximately 1 × 10^5^ transfected cells were inoculated in the upper chamber with serum-free medium (with a permeable membrane) and cultured for 24 h. Medium with 10% FBS was added into the lower chamber as a chemical inducer. After 12 h of incubation, cells that migrated to the bottom of the membrane were fixed with 4% formaldehyde for 10 min. After staining and fixation with 0.1% crystal violet, the cells were observed and counted under a microscope.

### Statistical analysis

All statistical analyses were performed using R 3.6.1. For quantitative values according to the normal distribution, Student’s t-test was used for comparison. For nonnormally distributed quantitative data, the Mann–Whitney U test was utilized for comparison. DFS was assessed with the Kaplan–Meier method, and the differences between the groups were compared by the log-rank test. *P* < 0.05 was considered statistically significant.

## Results

### Screening of DEGs

RNA-Seq data from the TCGA–CHOL dataset were downloaded from the TCGA database. Seven hilar/perihilar cholangiocarcinoma tissues and two distal cholangiocarcinoma tissues were excluded in accordance with the postoperative pathological results. The RNA-Seq data for 30 iCCA tissues and eight adjacent normal tissues were retained for subsequent analysis. The clinicopathological characteristics of the aforementioned 30 iCCA patients are listed in Table 1. After data preprocessing, 1019 DEGs (184 upregulated and 835 downregulated) were screened using the threshold criteria of FDR < 0.01 and |log2FoldChange|> 1.0 (Additional file 1: Table S1).

**Table 1:** Clinicopathological characteristics in two cohors

Characteristics	TCGA cohort (n = 29)	Validation cohort (n = 30)	*P* value
Age (years)			
≤ 50	5 (17.2)	7 (23.3)	0.561
> 50	24 (82.8)	23 (76.7)	
Gender			
Female	16 (55.2)	10 (33.3)	0.091
Male	13 (44.8)	20 (66.7)	
Family history			
Yes	17 (58.6)	–	–
No	11 (37.9)	–	
Hepatitis virus			
Yes	1 (3.4)	15 (50.0)	< 0.001
No	28 (96.6)	15 (50.0)	
CA19-9			
IU/mL	39.2 (21.5, 223.0)	16.9 (12.3, 36.5)	0.008
Pathologic T			
T 1	18 (62.1)	11 (36.7)	0.089
T 2	9 (31.0)	13 (43.4)	
T 3	2 (6.9)	2 (6.7)	
T 4	0	4 (13.3)	
Pathologic N			
N 0	22 (75.9)	15 (50.0)	0.001
N 1	3 (10.3)	15 (50.0)	
N X	4 (13.8)	0	
Pathologic M			
M 0	25 (86.2)	30 (100.0)	0.052
M 1/ M X	4 (13.8)	0	
Tumor TNM stage			
Stage I	18 (62.1)	2 (6.7)	< 0.001
Stage II	8 (27.6)	9 (30.0)	
Stage III	3 (10.3)	19 (63.3)	
Stage IV	0	0	
MVI			
Yes	4 (13.8)	5 (16.7)	1.000
No	25 (86.2)	25 (83.3)	
ESR1 expression			
Copies	5.9 (4.8, 7.0)	3.4 (2.3, 8.0)	0.074
Recurrent status			
Yes	17 (58.6)	17 (56.7)	1.000
No	12 (41.4)	13 (43.3)	
Survival status			
Alive	15 (51.7)	16 (53.3)	1.000
Dead	14 (48.3)	14 (46.7)	

TNM stage: ACJJ 6TH or 7TH TNM stage; Hepatitis virus: Hepatitis B and/or hepatitis C viruses; MVI: microvascular invasion

### Construction of the weighted gene coexpression network

**Fig. 1:**
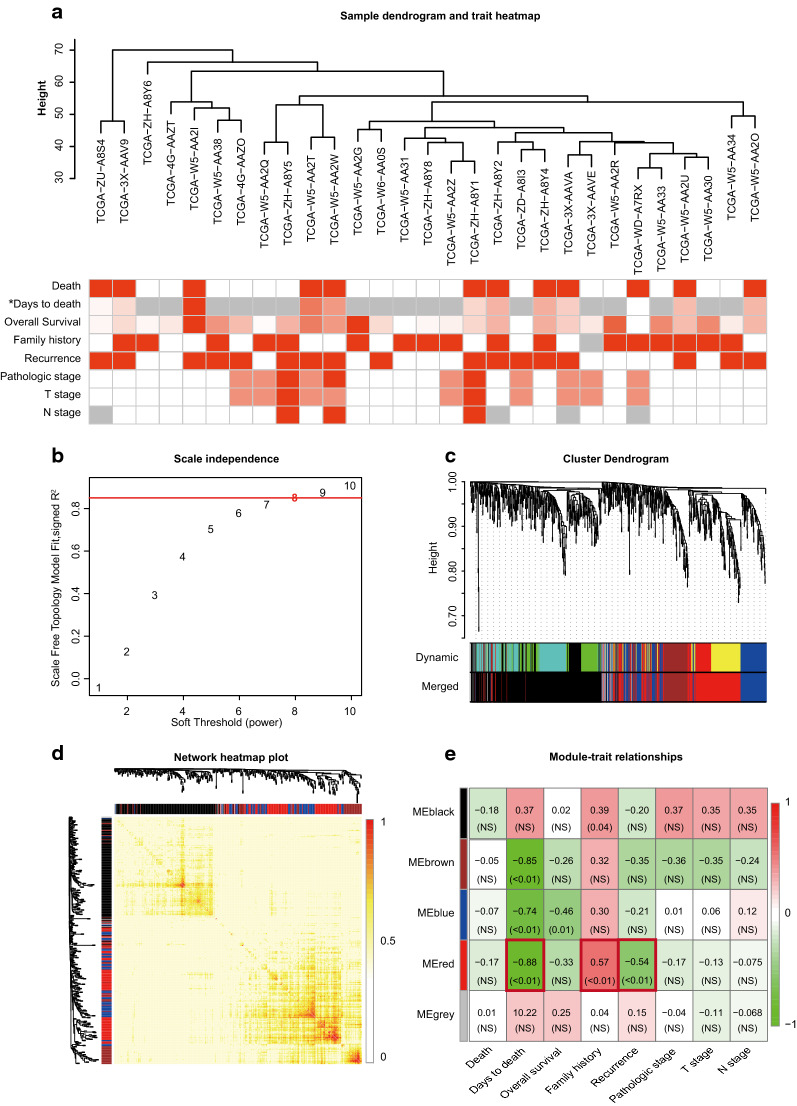
Construction of the weighted gene coexpression network. **a** Clustering dendrogram of 29 tumor samples and heatmaps of clinical traits. The color intensity in the heatmaps was proportional to increased days to death, improved overall survival, a higher pathological stage, higher T stage, or higher N stage. White indicates no death, no family history, or no recurrence, and red denotes a deceased patient who had tumor-related death, family history, or tumor recurrence. Gray represents values that are unavailable in the TCGA database; **b** Analysis of the scale-free fit index for various soft-thresholding powers (b); b = 8 was selected for subsequent analysis. **c** Clustering dendrogram of DEGs with dissimilarity based on topological overlap, together with assigned module colors. **d** Visualization of the gene network by using a heatmap. The heatmap depicts the TOM among all genes in the analysis. A light color represents low overlap, and a progressively darkening red color represents increased overlap. The gene dendrogram and module assignment are also presented along the left side and the top. **e** Module-trait heatmap. Each row corresponds to an ME, and each column corresponds to a clinical trait. Each cell contains the corresponding correlation and *P*-value; *NS* means *P* > 0.05

All DEGs were used to construct a weighted gene coexpression network. One sample was excluded because it was identified as defective according to the given criteria. The 29 remaining iCCA samples were clustered using the “flashClust” R package. The results of the clustering and relative trait analyses are illustrated in Fig. 1a. Then, thresholding powers from 1 to 10 were tested separately to identify the most appropriate soft-thresholding power. As shown in Fig. 1b, a power value of 8 was ultimately selected for constructing a scale-free network with a scale-free R^2^ of 0.87 and a mean connectivity of 10.12 (Additional file 2: Figure S1A). Seven coexpression modules were constructed after excluding the gray module. The MEDissThres value was set as 0.24 to merge similar modules, leaving four modules (Fig. 1c, Additional file 2: Figure S1B). The network heatmap of the four modules is shown in Fig. 1d, and the results showed high correlations between the intramodular genes. The black, blue, brown and red modules included 406, 171, 132 and 231 genes, respectively (Additional file 3: Table S2).

### Identification of the key module

The interaction relationship between the four modules was calculated. All these modules independently validated each other, indicating a high level of independence between the four modules (Additional file 2: Figure S1C). In addition, the correlation between the module eigengenes and clinical traits was calculated (Fig. 1e). The red module was selected as the key module because it had the three highest correlations with the day to death, family history and recurrence status. We were particularly interested in the relationships between the recurrence status of the patients and the expression of hub genes in the red module, given its potential significance in the prognosis of iCCA patients. Furthermore, univariable COX regression for DFS was performed, and the results revealed that ESR1 expression was an independent prognostic factor for better DFS of iCCA patients in both cohorts (Table 2).

**Table-2:** Univariable Cox regression for DFS in two cohorts

Characteristics	*P-value*	HR	Low 95% CI	High 95% CI
TCGA cohort				
Age, > 50 vs. ≤ 50	0.785	0.805	0.170	3.810
Gender, Male vs. Female	0.279	0.514	0.154	1.713
Family history, Yes vs. No	0.109	0.384	0.119	1.240
Hepatitis virus infection, Yes vs. No	0.535	0.043	0.00	874.9
CA19-9 level	0.521	1.002	0.997	1.006
Tumor TNM stage	0.376	1.832	0.480	7.001
MVI, Yes vs. No	0.964	0.953	0.121	7.496
ESR1 expression	**0.031**	0.594	0.370	0.953
Validation cohort				
Age, > 50 vs. ≤ 50	0.700	1.253	0.398	3.941
Gender, Male vs. Female	0.600	0.771	0.293	2.034
Family history, Yes vs. No	–	–	–	–
Hepatitis virus infection, Yes vs. No	0.837	0.904	0.346	2.363
CA19-9 level	0.066	1.002	1.000	1.003
Tumor TNM stage	0.619	0.835	0.409	1.702
MVI, Yes vs. No	**0.003**	5.527	1.790	17.066
ESR1 expression	**0.025**	0.804	0.665	0.972

The bold data mean that the characteristics are significant prognostic factors for DFS (*P* < 0.05)

### Functional enrichment analysis of genes in the red module

**Fig. 2:**
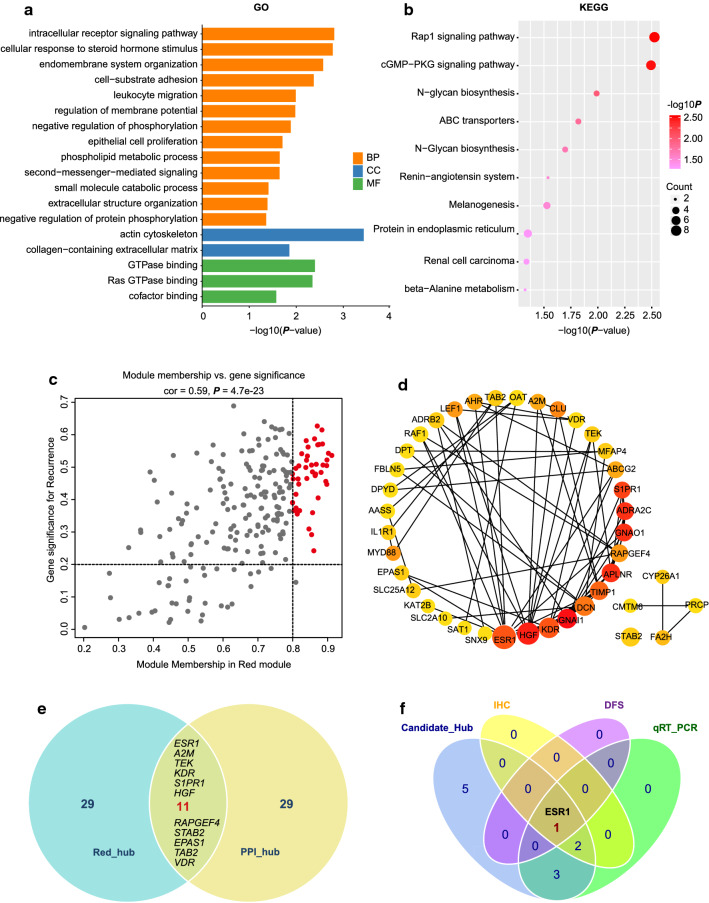
Detection and validation of real recurrence-related hub genes of iCCA. **a** GO enrichment analysis of the red module: all terms were analyzed (*P* < 0.05). **b** KEGG pathway enrichment analysis of the red module: the top 10 pathways were shown. **c** Scatterplot of module eigengenes in the red module. The red dots represent genes with MM > 0.8 and GS > 0.2, which composed the *Red_Hub* gene list. **d** The 40 key genes in the PPI network detected by the *cytoHubba* algorithm, which formed the *PPI_Hub* gene list. The rank of genes was indicated by color (the redder the color is, the more important the gene is), and the number of edges was indicated by the diameter of the circle (the greater the number of edges is, the larger the diameter is). **e** Detection of candidate hub genes: 11 genes (ESR1, A2M, TEK, KDR, S1PR1, HGF, RAPGEF4, STAB2, EPAS1, TAB2 and VDR) were selected as candidate hub genes. **f** Detection of real hub genes: three methods including qRT-PCR, IHC and DFS analysis were used to determine the real hub genes. ESR1 was selected to be the real hub gene

All genes in the red module were uploaded to DAVID for functional enrichment analysis. All significantly enriched GO terms (*P* < 0.05) are plotted in Fig. 2a. The GO enrichment analysis showed that the genes in the red module were significantly enriched in terms of cofactor binding, GTPase binding, negative regulation of protein phosphorylation, second messenger-mediated signaling, and epithelial cell proliferation. Most of these terms were related to tumorigenesis and tumor microenvironment regulation. Then, KEGG pathway enrichment of genes in the red module was performed (Fig. 2b). The genes in the red module were significantly enriched in pathways that were closely related to malignancy. These pathways included the Rap1 signaling pathway, cGMP-PKG signaling pathway, and N-glycan biosynthesis. In summary, these results indicate that genes in the red module are closely associated with tumor development and disease progression.

### Identification of candidate hub genes in the red module

The candidate hub genes in the red module were initially selected based on the following criteria: absolute value of GS for recurrence > 0.2 and MM > 0.8 (Fig. 2c). Forty genes were selected and comprised the *Red_Hub* gene list (Fig. 2e, Additional file 4: Table S3). A PPI network was constructed using the appropriate cutoff for the defective samples of confidence > 0.4 (Additional file 2: Figure S1D). The key genes in the PPI network were screened by employing the *cytoHubba* algorithm. The 40 genes were filtered and formed the *PPI_Hub* gene list (Fig. 2d). The genes common to the two gene lists were selected as candidate hub genes for predicting recurrence. Finally, 11 genes (Fig. 2e), namely, ESR1, A2M, TEK, KDR, S1PR1, HGF, RAPGEF4, STAB2, EPAS1, TAB2, and VDR, were selected. Further validation experiments were conducted for these candidate hub genes.

### Validation of real hub genes in the red module

**Fig. 3:**
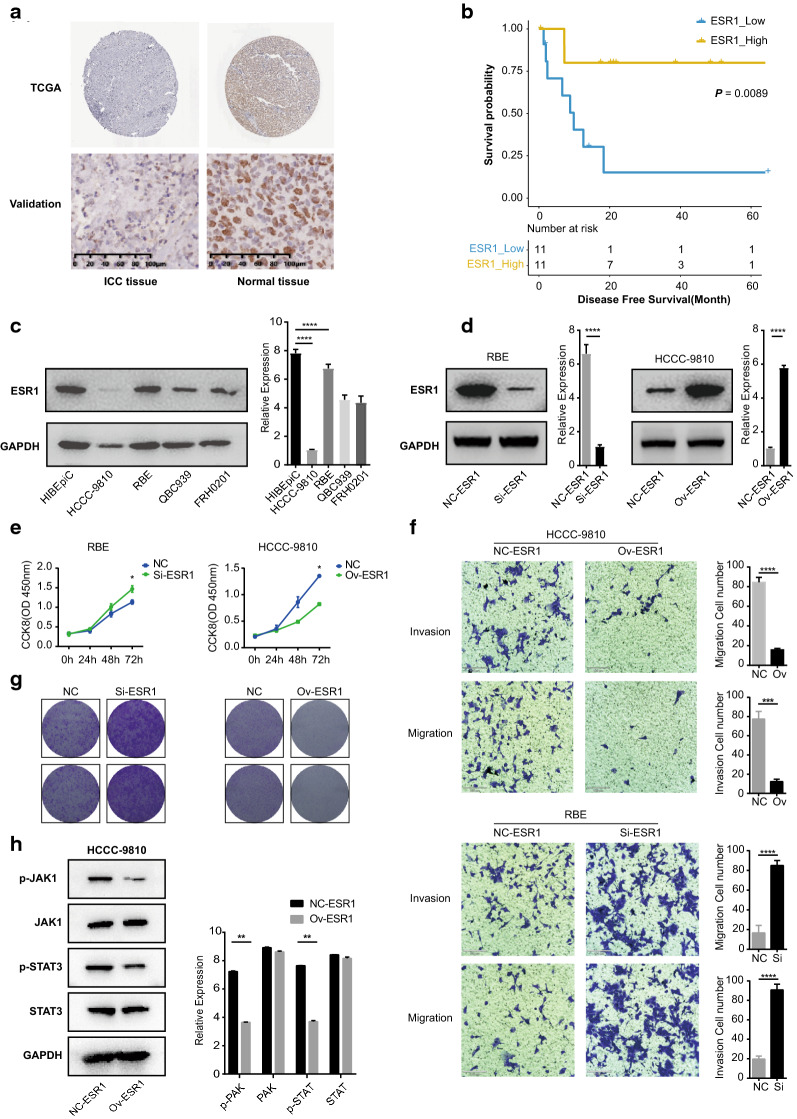
The effect of ESR1 on iCCA cell proliferation, migration and invasion. **a** The IHC results for ESR1 in iCCA tissues and normal liver tissues based on the Human Protein Atlas database and samples from the validation cohort. ESR1 was significantly overexpressed in normal liver tissue *vs.* iCCA tissue. **b** Survival analysis of the association between ESR1 expression level and DFS based on the TCGA database. Low ESR1 expression was associated with a poor prognosis in iCCA patients. **c** Comparison of ESR1 expression levels in iCCA cell lines (QBC939, RBE, HCCC-9810, and FRH0201) and a normal biliary epithelial cell line (HiBEpiC). The RBE cell line displayed the highest expression levels, and the HCCC-9810 cell lines displayed the lowest expression levels of the four iCCA cell lines. The HCCC-9810 cell line was chosen to conduct a gain-of-function experiment, and the RBE cell line was chosen to conduct a loss-of-function experiment. **d** The ESR1 expression level of the RBE cell line in the Si-ESR1 group and NC-ESR1 group. The ESR1 expression level of HCCC-9810 cells in the Ov-ESR1 group and the NC-ESR1 group. **e** CCK-8 assay of RBE and HCCC-9810 cells. **f** Transwell assay of HCCC-9810 and RBE cells. **g** Colony formation assay of RBE and HCCC-9810 cells. **h** The expression of essential molecules in the JAK/STAT3 signaling pathway after upregulating the expression of ESR1 in the HCCC-9810 cell line

Three approaches were used to screen the real hub genes. First, qRT-PCR was adopted to measure the relative transcript abundance of the 11 candidate hub genes between 30 iCCA tumor tissues and adjacent nontumor tissues from patients in the validation cohort. Four genes were excluded from this step because the difference in the relative mRNA expression levels between the tumor tissues and adjacent nontumor tissues was not statistically significant (*P-*value > 0.05). Seven candidate hub genes (ESR1, A2M, RAPGEF4, S1PR1, HGF, STAB2, and VDR) were retained for the following validation (Additional file 2: Figure S1E). The IHC results from the Human Protein Atlas database demonstrated that only three genes (ESR1, A2M, and RAPGEF4) showed expression differences at the protein level between the iCCA specimens and normal liver specimens (Fig. 3a, Additional file 2: Figure S1E). Then, the Table-S4 values of ESR1, A2M, and RAPGEF4 were determined using receiver operating characteristic (ROC) analysis and Youden index calculation. ESR1 was filtered and deemed the real hub gene that has a significant impact on the DFS of the patients (Fig. 3b, Additional file 5: Table S4).

### The protein expression level of ESR1 in cholangiocarcinoma cell lines

To detect the protein expression level of ESR1 in cholangiocarcinoma cell lines (QBC939, RBE, HCCC-9810, and FRH0201) and a normal biliary epithelial cell line (HiBEpiC), a Western blot assay was conducted. The results showed that the ESR1 expression level in the HCCC-9810 cell line was the lowest and that the RBE cell line was the highest among the four cholangiocarcinoma cell lines (Fig. 3c). Therefore, we conducted gain-of-function experiments in the HCCC-9810 cell line and loss-of-function experiments in the RBE cell line. As shown in Fig. 3d, the protein expression level of ESR1 in the Si-ESR1 group was significantly lower than that in the NC-ESR1 group in the RBE cell line (*P* < 0.0001), and the protein expression level of ESR1 in the ESR1 overexpression (Ov-ESR1) group was significantly higher than that in the NC-ESR1 group in the HCCC-9810 cell line (*P* < 0.0001).

### The effect of ESR1 on iCCA cell proliferation, migration and invasion

To determine the effect of ESR1 on iCCA cell proliferation, CCK-8 and colony forming assays were performed. ESR1 overexpression significantly inhibited cell proliferation, while ESR1 knockdown promoted cell proliferation (Fig. 3e, g). To further determine the effect of ESR1 on cell migration and invasion, a Transwell assay was performed. ESR1 overexpression markedly inhibited the migration and invasion abilities of HCCC-9810 cells, and knockdown of ESR1 promoted the migration and invasion abilities of RBE cells (*P* < 0.001, Fig. 3f). All these findings demonstrate that ESR1 expression inhibits the proliferation, migration and invasion of iCCA cells.

### ESR1 functions as a tumor suppressor by inhibiting the JAK/STAT3 signaling pathway

To further investigate the potential antitumor mechanism of ESR1, we explored whether ESR1 inhibits the proliferation, migration and invasion of iCCA cells through the JAK/STAT3 signaling pathway. As the experiments illustrated, ESR1 overexpression resulted in a significant reduction in the expression of p-JAK1 and p-STAT3 in the HCCC-9810 cell line, while the expression levels of JAK1 and STAT3 did not change significantly. Our results illustrated that ESR1 may act as a tumor suppressor by inhibiting the JAK/STAT3 signaling pathway.

## Discussion

iCCA is the second most common primary liver cancer and has a high likelihood of postoperative recurrence. The incidence of iCCA is on the rise worldwide [16]. High-throughput genomic analysis is a promising tool that has considerable clinical applications in medical oncology [17, 18]. The initiation and progression of iCCA are complex, given that they involve various genetic changes [19]. Although many genes have been selected for the diagnosis and treatment of iCCA, novel potential biomarkers are still required to improve the understanding of tumor progression, especially tumor recurrence after surgery. In this study, WGCNA was used to screen candidate biomarkers associated with the recurrence of iCCA.

First, based on the TCGA-CHOL dataset, 1019 DEGs were filtered out after dataset preprocessing and were used to construct a weighted gene coexpression network. Although our threshold for filtering DEGs was higher than that in previous similar studies [13, 17], it proved to be effective. After network construction, module identification, and assessment of the relations between the modules and clinical traits, we found that the red module was associated with tumor recurrence. Finally, after a series of hub gene identification and validation studies, ESR1 was selected as the real hub gene in our analysis and was significantly associated with recurrence.

ESR1 encodes an estrogen receptor. Estrogen and its receptors are essential for sexual development and reproductive function. Previous studies have shown that ESR1 is closely related to the occurrence and development of various urogenital cancers [20–23], especially breast cancer [24, 25] and endometrial cancer [26–28]. Recent studies have reported the important role of ESR1 in non-small-cell lung cancer (NSCLC) [29, 30] and bladder cancer [31]. In addition, another study demonstrated that the methylation ratio of ESR1 was closely associated with old age and smoking history in patients with NSCLC [30].

Recently, with the rapid development of genome-wide technologies, we have gained deep insight into the molecular mechanisms of iCCA, and more potential biomarkers have been identified for clinical use. For example, Chen et al. found that TGF-β1 was an independent risk factor for early tumor recurrence in iCCA [32]. In addition, a study found that iCCA patients with high levels of CYFRA21-1 had worse 3-year recurrence-free survival rates than those with low levels (DFS: 25.0% *vs*. 76.2%, P < 0.01), which suggests that CYFRA21-1 is associated with tumor recurrence [33]. Other studies showed that low expression of SMAD4, a tumor suppressor protein, increased the rates of lymph node recurrence in iCCA [34]. High epidermal growth factor receptor (EGFR) expression was also identified as a risk factor for poor overall survival (*P* = 0.0006) and recurrence (*P* = 0.0335) in iCCA patients [35].

In 2020, using data from the Gene Expression Omnibus (GEO) database, Qin et al. found that ESR1 was downregulated in iCCA patients. ESR1 and its corresponding miRNA, hsa-miR-26a-5p, might be novel biomarkers for prognosis [36]. However, Qin et al. failed to validate their conclusions through in vitro experiments and clinical tissue samples. Moreover, ESR1 was identified through an indirect method in Qin’s study. In this study, we found that ESR1 is a hub gene in iCCA that is significantly associated with recurrence through WGCNA and PPI network analysis. Then, a series of in vitro experiments demonstrated that ESR1 negatively regulates iCCA cell proliferation, migration and invasion. Furthermore, we performed in-depth studies to understand the mechanism and illustrated that ESR1 may act as a tumor suppressor by inhibiting the JAK/STAT3 signaling pathway. Our results suggest that the roles of ESR1 in human cancers are more extensive than previously believed and deepen our understanding of ESR1 function in human cancers.

Our study has several limitations. First, ESR1 was identified as a hub gene through analysis of 29 iCCA patients from the TCGA database, and the validation cohort was also small, with only 30 patients; thus, the statistical power of the calculations in our study is low. More iCCA patients should be used to validate our conclusions in the future. Second, our study was a retrospective study, so the significance and robustness of the results and recurrence-related hub genes should be validated in prospective cohorts. Last, the clinical applicability of ESR1 as a biomarker is limited due to a lack of adequate reproducibility in daily clinical practice.

In conclusion, our research revealed that ESR1 plays a critical role as a tumor suppressor in iCCA. ESR1 significantly impacts the prognosis of iCCA patients and markedly inhibits cell proliferation, migration and invasion. ESR1 may exert a tumor suppressor function by inhibiting the JAK/STAT3 pathway. The findings of the present study may help researchers gain a better understanding of the mechanism of iCCA development and provide a novel target for the treatment of iCCA in the future.

## Conclusion

1. ESR1 was identified as a recurrence-related gene of iCCA via WGCNA and PPI network analysis.
2. Low expression of ESR1 predicts a poor prognosis in iCCA patients.
3. The expression of ESR1 inhibits the proliferation, migration and invasion of iCCA cells.
4. ESR1 may exert a tumor suppressor function by inhibiting the JAK/STAT3 signaling pathway.

## Supplementary Information

**Additional file 1: Table-S1.** The list of the 1019 DEGs in 29 iCCA samples from TCGA.

**Additional file 2: Figure S1. (A)** Analysis of the mean connectivity for various soft-thresholding powers (b). Mean connectivity (y-axis) is a strictly decreasing function of the power b (x-axis). **(B)** Clustering of MEs. MEDissTres was set as 0.24 to merge similar modules. The red and yellow modules were merged into the new red module, which was selected as the hub module in our research. **(C)** Heatmap of the adjacencies in the hub gene network. **(D)** PPI network of all genes in the red module. **(E)** qRT-PCR results for ESR1, A2M, S1PR1, HGF, VDR, RAPGEF4 and STAB2. **(F)** IHC results for A2M and RAPGEF4 in iCCA tissues and normal liver tissues based on the Human Protein Atlas database. A2M and RAPGEF4 were significantly overexpressed in normal liver tissue *vs.* iCCA tissue.

**Additional file 3: Table-S2.** GS and MM in four integrated modules.

**Additional file 4: Table-S3.** The genes in red_hub list and PPI_hub list.

**Additional file 5: Table-S4.** Validation of real hub genes.

## Data Availability

All data generated or analyzed during this study are included in the additional files.

## Abbreviations

iCCA: Intrahepatic cholangiocarcinoma
DEGs: Differentially expressed genes
TCGA: The Cancer Genome Atlas
PPI: Protein–protein interaction
GO: Gene ontology
KEGG: The Kyoto Encyclopedia of Genes and Genomes
HCC: Hepatocellular carcinoma
TK: Tyrosine kinase
GS: Gene significance
MS: Module significance
ME: Module eigengene
DAVID: The Database for Annotation, Visualization, and Integrated Discovery
STRING: The search tool for the retrieval of interacting genes database
IHC: Immunohistochemistry
GEPIA: Gene expression profiling interactive analysis
SDS: Sodium dodecyl sulfate
PAGE: Polyacrylamide gel electrophoresis
ECL: Enhanced chemiluminescence
DFS: Disease-free survival
NSCLC: Non-small-cell lung cancer
