# Supplementary material for: ESR1 as a recurrence-related gene in intrahepatic cholangiocarcinoma: a weighted gene coexpression network analysis
Source: Cancer Cell Int. 2021 Apr 17;21:225. doi: 10.1186/s12935-021-01929-5 (PMC8052670; doi:10.1186/s12935-021-01929-5)
Supplement: Supplementary file 2 — Additional file 2: Figure S1. (A) Analysis of the mean connectivity for various soft-thresholding powers (b). Mean connectivity (y-axis) is a strictly decreasing function of the power b (x-axis). (B) Clustering of MEs. MEDissTres was set as 0.24 to merge similar modules. The red and yellow modules were merged into the new red module, which was selected as the hub module in our research. (C) Heatmap of the adjacencies in the hub gene network. (D) PPI network of all genes in the red module. (E) qRT-PCR results for ESR1, A2M, S1PR1, HGF, VDR, RAPGEF4 and STAB2. (F) IHC results for A2M and RAPGEF4 in iCCA tissues and normal liver tissues based on the Human Protein Atlas database. A2M and RAPGEF4 were significantly overexpressed in normal liver tissue vs. iCCA tissue. [file 12935_2021_1929_MOESM2_ESM.pdf]

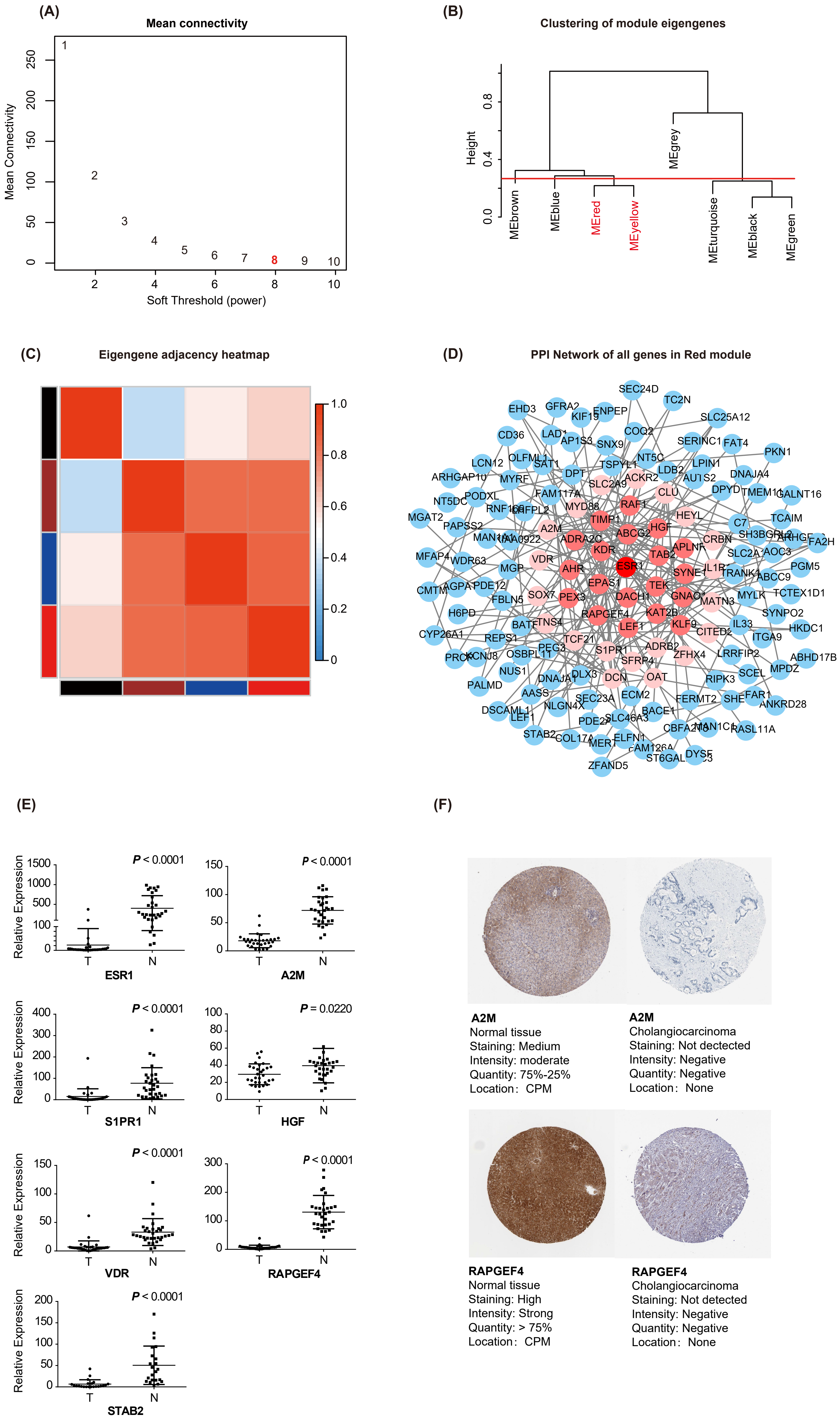
(A)
Mean connectivity
Mean Connectivity
Soft Threshold (power)
(B)
Clustering of module eigengenes
Height
MEbrown
MEblue
MEred
MEyellow
MEgrey
MEturquoise
MEblack
MEgreen
(C)
Eigengene adjacency heatmap
(D)
PPI Network of all genes in Red module
(E)
Relative Expression
P < 0.0001
ESR1
A2M
S1PR1
P = 0.0220
HGF
VDR
RAPGEF4
STAB2
T
N
(F)
A2M
Normal tissue
Staining: Medium
Intensity: moderate
Quantity: 75%-25%
Location： CPM
A2M
Cholangiocarcinoma
Staining: Not dectected
Intensity: Negative
Quantity: Negative
Location： None
RAPGEF4
Normal tissue
Staining: High
Intensity: Strong
Quantity: > 75%
Location： CPM
RAPGEF4
Cholangiocarcinoma
Staining: Not detected
Intensity: Negative
Quantity: Negative
Location： None
